# "The Hospital" Nursing Mirror

**Published:** 1899-11-18

**Authors:** 


### "The Hospital" Nursing Mirror.

Being the Nursing Section of "The Hospital."

[Contributions for this Section of "The Hospital" should be addressed to the Editor, The Hospital, 28 & 29, Southampton Street, Strand, London, W.C., and should have the word "Nursing" plainly written in left-hand top corner of the envelope.]

### Notes on News from the Nursing World.

### THE "PRINCESS OF WALES" HOSPITAL SHIP.

We have received a number of letters from our readers desiring to contribute to the fund for the "Princess of Wales" hospital ship. At present it is not necessary to invite contributions; but later on, if occasion requires, a notification will be made.

### THE PRINCESS OF WALES AND THE NURSES FROM THE LONDON HOSPITAL.

The two nurses from the London Hospital—Miss Morgan (matron's assistant) and Nurse Dickenson—who went to Copenhagen at the desire of the Princess of Wales in order to see how Dr. Finsen's light apparatus was used in the treatment of lupus, speak in the warmest manner of the kindness shown to them while they were in the Danish capital. They devoted about five hours every morning to the work, which, though rather trying at first, soon became interesting, and they describe [illegible] arrangements as admirable, every detail of treatment and nursing being well thought out. The Princess of Wales, with other members of the Danish Royal family, visited Dr. Finsen's hospital, and showed much interest in the English nurses, telling them how pleased she was to know that the new treatment was going to be tried at the "London." The nurses at the hospital were most friendly.

### ALLOCATION OF THE NURSING SISTERS FOR SOUTH AFRICA.

The following are the names of the nursing sisters proceeding to South Africa and the units on the lines of communication to which they are allotted:—

No. 1 General Hospital.—Superintendent Miss A. Garriock; Nursing Sisters Miss S. Y. Snowdon, Miss A. A. Murphy, Miss A. Nixon, Miss A. C. L. Anderson, Miss H. L. Neale, Miss A. R. Rose-Innes, Miss A. Guthrie, and Miss M. Lowe.*

No. 2 General Hospital.—Superintendent Miss L. Hardement; Nursing Sisters Miss R. T. MacIntyre, Miss I. A. G. Kinahan, Miss M. L. de Montmorency, Miss A. Barker, Miss E. Maclean, Miss E. M. Beck, Miss L. M. Culverwell, and Miss P. Watson.*

No. 3 General Hospital.—Superintendent Miss S. J. Browne; Nursing Sisters Miss L. E. Steen, Miss M. Wilson, Miss E. A. Cox, Miss W. Potter, Miss D. Palmer, Miss M. C. Rose, Miss M. E. Harding, and Miss E. M. Whiteman.*

No. 4 General Hospital.—Superintendent Miss M. C. Cole; Nursing Sisters Miss C. H. Potts, Miss M. C. S. Knox, Miss C. H. Keer, Miss E. M. Todd, Miss M. Mark, Miss H. F. Young, Miss L. M. Todd, and Miss E. Laughton.*

No. 1 Hospital Train.—Nursing Sisters Miss M. Falcon* and Miss E. Sainsbury.*

No. 2 Hospital Train.—Nursing Sisters Miss M. L. T. Babb* and Miss L. R. Woollcombe.*

No. 1 Sick Transport (ss. "Spartan," Admiralty Transport No. 11).—Nursing Sisters Miss G. E. Saunder, Miss D. D. Tripp, and Miss M. R. Makepeace.

No. 2 Sick Transport (ss. "Trojan," Admiralty Transport No. 10).—Nursing Sisters Miss A. E. Tait, Miss C. K. E. Steel, and Miss L. B. A. Drury.

(* Army Nursing Service Reserve.)

All appointments, however, are subject to modification by the orders of the principal medical officer with the Commander-in-Chief in South Africa.

### NURSING THE WOUNDED AT WYNBERG.

It is a satisfaction to know that Wynberg, where some of our wounded in the South African war are being nursed, and are doing well, is beautifully situated in a forest of pine and gum trees. The hospital at Wynberg is small, with a matron, three staff nurses, and two probationers; the average number of beds occupied is sixteen. There is ordinarily no lack of room, but in the present circumstances the adjoining barracks are being used as a base hospital. Even the 500 beds to be thus provided will doubtless be insufficient, and the nursing staff will be supplemented by some of the Army and Army Reserve Sisters who have been sent out from England.

### THE HOSPITAL SHIP "MAINE."

At the first meeting of the General Committee of the American Hospital Ship Fund, presided over by Lady Randolph Churchill, who made an extremely eloquent little speech on the value of deeds in cementing the friendship between England and America, it was stated that the quarters in the "Maine" for the lady nurses are in the after portion of the vessel around the main saloon. Miss Hibbard, superintendent of the United States Government Hospitals, is to be chief superintendent of nurses on board. Accompanying her from New York in the "Mesaba," which is now on her way to this country, are ten male as well as four female nurses.

### HOW NURSES MAY HELP OUR SOLDIERS.

The Secretary of the Trained Nurses' Club asks us to make known that it has been arranged to receive at 12, Buckingham Street, Strand, any small contributions which nurses may care to make in the way of games, &c., and to have these forwarded to the troops in South Africa for the use of the sick and wounded. Many individual nurses may possess spare "Halma," draughts, or other games, which they would like to give, but are deterred by thinking it not worth while to forward so small a contribution to the large societies which are collecting for our soldiers. Here, then, is an opportunity for making use of "little things." Those who cannot do much may yet materially assist by supplying Tommy Atkins with entertainment during his convalescence from sickness or wounds. Nurses well know how recovery is aided by the judicious provision of occupation and amusement.

### ENGLISH NURSES IN INDIAN PLAGUE HOSPITALS.

Under the heading of "An Invasion of Nurses," the *Times of India* attacks the policy of the Indian Government in dispatching a number of nurses for plague duty in the Bombay Presidency, and suggests that the English nurses are not popular among the natives. A striking commentary on the suggestion is furnished by the telegraphic report on Monday that, after Lord Curzon had visited the hospitals at Poona, he was photographed in a group with the nurses and plague patients from the convalescent wards, amid the utmost enthusiasm. If there had been any feeling either against the Viceroy or the English nurses, the natives would scarcely have "almost smothered Lord Curzon" with garlands of flowers. It is asked, however, whether "these nurses, who are strangers in a strange land, are better able to cope with the disease than the Anglo-Indian, Eurasian, and native nurses?" There are always people prepared to stand up for vested interests, and we quite recognise the feeling which leads some people to claim this position for local nurses, but the justification for sending out to Bombay some of the best of English nurses is that they have enjoyed a kind of training and experience which have not been enjoyed by Eurasians or natives, and which have been found to be of the greatest value in nursing plague patients. "What we want for these cases," says a critic in the columns of an Anglo-Indian contemporary, "is the compassionate nurse." But surely it is better that the compassionate nurse should be as fully equipped as possible by her training to tend the sufferers from the disease; and no one ventures to hint that any lack of compassion has been manifested by the brave English ladies who undertake plague service in India

### DUBLIN SCHOOL FOR NURSES.

To Dublin belongs the honour of inaugurating a very helpful step in the training of nurses. There is no difficulty in arranging classes for the probationers at large hospitals, but in small institutions it is a great tax. It sometimes happens that whilst the young nurses are well taught the practical work the theoretical branches are rather neglected. The arrangement to provide suitable courses of lectures by co-operation such as has been made by the "Dublin Metropolitan Technical School for Nurses" is therefore sensible, and should be serviceable. The institutions combining are, so far, Usher's Quay Nursing Institution, the Dublin Orthopædic Hospital, the Richmond, Whitworth, and Hardwicke Hospitals, and Sir Patrick Dun's Hospital. The governing body is composed of the matrons and one representative of the medical staff of each co-operating institution, and is under the patronage of the presidents of the Royal Colleges of Physicians and Surgeons in Ireland. The year is divided into three terms, and six lectures are delivered in duplicate during each term. The lectures during the first term are to be on anatomy and surgical nursing; in the second on physiology and medical nursing; and in the third on hygiene and invalid cookery. As soon as a student earns the certificate of her training school the diploma of the technical school is also given to her when she satisfies the examiners. It is a distinct qualification. The co-operating institutions subscribe at a definite rate per probationer, to pay the lecturer and the expenses connected with the study and examinations; but outside students may share the same privileges if they wish to do so at slightly higher fees.

### RESIGNATION OF SISTER BUXTON.

The prospect of the early retirement of Sister Buxton from the London Hospital is deeply regretted by the matron, and by every member of the nursing staff. It is more than 37 years since Miss Buxton first entered the hospital, and she has been under three matrons. In the course of an interesting account of her career, the *London Hospital Gazette* says:—

"She assisted Miss Swift to prepare the newly-erected Queen Victoria wards for the reception of the Queen. She was honoured on that occasion by a visit from her Majesty. The authorities had not included Buxton ward in the route to be traversed by the Queen, and it was thought unnecessary to decorate it. But it is said that a hint reached her Majesty respecting a small patient who was heart-broken at the prospect of not seeing her. With her usual kindness, she thereupon expressed her decision to go into the the ward in which the loyal little patient lay—so that Buxton had the honour of a longed-for, but little-expected, special visit. When Miss Lückes entered on her duties her path was no easy one; but she found in Sister Buxton an absolutely loyal worker, one who, from that day to this, has upheld authority and set an example of honourable integrity. By her plain straightforwardness Sister Buxton has ever gained respect; by her knowledge and vast experience she has earned a reputation for exact observation—which many must envy her."

Before she joined the staff at the "London," Sister Buxton was a nurse at the Great Ormond Street Hospital for Sick Children.

### THE NURSING IN BIRMINGHAM INFIRMARY.

In reference to our note on the training of nurses at Birmingham Infirmary, it is pointed out by the matron of the infirmary that only the practice of training fee-paying nurses has been relinquished. Miss Gibson says, "We shall continue to train as we have always done three-yearly probationers, the only difference being that we can now train a larger number for three years than we could whilst we took paying pupils. I consider that this is a great gain." There is no room for doubt upon the subject, and the action of the managers of the Birmingham Infirmary will, it may be hoped, be followed by other important bodies.

### NEVER BE ASHAMED OF YOUR AGE.

A severe lesson has been read to their nurses by the Scarborough Board of Guardians. The House Committee, in reporting on the ages of the new nurses, found out that one of them had understated her age by six years, and the other by one year. In the latter case a satisfactory explanation seems to have been given; but the Board decided to ask the former to resign. The moral of this incident is obvious. Even if an appointment is obtained by under-stating age, no good results can come of such tactics, and, as this case shows, the chances of the discovery of the imposition are greater than the chances of the concealment of the truth.

### PROBATIONERS AND THE POSITION OF SUPERINTENDENT NURSE IN WORKHOUSES.

The Nursing Committee of the Swansea Board of Guardians are agitating for the modification of the order of the Local Government Board under which no probationer in a workhouse where there is no resident medical man can take the position of superintendent nurse at the end of her three years' service. They have accordingly induced the Guardians who sit on the Hospital Committee to allow the probationers at the workhouse to take the course of lectures with the hospital nurses and sit at the annual examinations, in the hope that, under these circumstances, the former will be considered fully-qualified nurses at the end of their three years' service. The decision of the Local Government Board will be awaited with interest in the nursing world.

### COMPLIMENTS TO THE LADY SUPERINTENDENT OF CHELTENHAM NURSING ASSOCIATION.

The proceedings at the eighteenth annual meeting of the Cheltenham District Nursing Association were of a very gratifying character, and a remarkable compliment was paid by Mr. Nelson Foster, the hon. treasurer, to the lady superintendent, Miss Bladen. Commenting on the fact that all our present time and thoughts were absorbed and centred in South Africa, where our noble soldiers are upholding the best traditions of the British army, Mr. Foster said: "Were it possible to transfer their activities. Miss Bladen would make another Florence Nightingale." The Chairman of the meeting, Dr. E. T. Wilson, in moving the adoption of the report, observed: "All the members of the staff were thoroughly qualified by training for their work, and all, from the lady superintendent down, brought to the discharge of their duties a combination of heart and brain and hand such as perhaps was not seen in an equal degree in any other class of work. Of the superintendent he could scarcely venture to say anything, as she would not like to be reprimanded in public for overworking herself." It was subsequently mentioned that the medical profession in Cheltenham are unanimous in their support of the district nursing.

### ENGLISH NURSES AND WELSH PATIENTS.

At a meeting of the Bangor and Beaumaris Board of Guardians, prior to the appointment of a new head nurse, the Chairman stated that neither of the only two applicants for the position could speak Welsh. Thereupon, the Rev. Evan Evans, of Bangor, declared that they both laboured under a serious disadvantage. This was to some extent admitted by the Chairman, but he intimated that as the Guardians had been vainly trying to obtain a head nurse for three months, it was a matter of Hobson's choice. It was, however, reserved for the master of the workhouse to settle the question. In reply to the Chairman, he said that "an English nurse had been engaged at the hospital, and no difficulty was experienced regarding her ignorance of the Welsh language." After such a practical contribution to the discussion even the Rev. Evan Evans did not press his objection to English nurses nursing Welsh patients.

### DIFFICULTIES AT BRIDGWATER.

The Bridgwater Board of Guardians have appointed a committee to inquire into certain complaints made by the head nurse at the hospital against the master and officials of the workhouse. In the discussion which took place on the subject, Mrs. Harman, one of the Guardians, stated that it was not a question of a particular nurse complaining. "Ever since she had been a Guardian every nurse that had left had told her the same thing—that they could not get on with the house side. There was nothing definite, but a constant worrying going on." Mrs. Harman added that the Guardians only knew too well the difficulty they had to get nurses, "but the difficulty to keep them was greater." On the other hand, the master declares that he has never said an unkind word to the head nurse since she has been there, and that the other nurses have complained to him about her severity. Whatever may be the actual facts of the case, it is obvious that so long as feeling of this kind prevails there must continue to be difficulties about the nursing under the Bridgwater Guardians.

### THE NORFOLK AND NORWICH HOSPITAL.

The recent anxiety felt by the management of the Norfolk and Norwich Hospital is abating. A little time ago it was feared that a ward would have to be closed, but owing to the liberality of the subscribers such a misfortune is not now likely to happen. Mr. Colman most generously doubled his annual subscription, and suggested that many other contributors should do the same. Among those who have liberally responded to the appeal are Lord and Lady Leicester. This is another instance of the munificence of the president of the hospital and his wife. Lord Leicester, in a letter to a local paper, says: "It would indeed be sad if any of the wards were closed for want of funds, or that any portion of the invested capital of the hospital should be realised to maintain its annual expenditure."

### NURSING THE POOR IN DUNDEE.

Some very nice things were said by the speakers at the annual meeting of the subscribers and friends of the Dundee Sick Poor Nursing Society about their nurses, to whose kindness of heart, ability, tact, and devotion to duty, the report bore eloquent testimony, Dr. Alexander Campbell, on behalf of the medical profession, said that they wished it to be known how much they appreciated the work of the society. "There was no more deservedly popular class in Dundee than the district nurses. They did their work most faithfully; it was truly valued by the sick poor and their friends, and by no one more appreciated than by the general practitioner." Subsequently, Dr. Campbell made an appeal to the public spirit of Dundee, and suggested that the classes to whom the nurses often ministered could afford to make some contribution to the nursing association for services received. That is the case in a great many places besides Dundee, though we are afraid that the contribution is seldom forthcoming.

### SHORT ITEMS.

The "Carisbrook Castle" has arrived at Cape Town. The passengers included Mrs. Richard Chamberlain, Miss Chamberlain, Colonel Young, of the Red Cross Society, and Nurses Hardement, Culverwell, Watson, Falcon, Babb, Woollcombe, Maclean, Beck, Kinahan, Sainsbury, de Montmorency, Barker, and Macintyre.—The Hamilton Association for Providing Trained Male Nurses will keep open the places of those of their nurses who have been transferred from the Reserve to the colours for service with the Royal Army Medical Corps in South Africa.—At the second annual meeting of the Reading branch of the Queen Victoria Nursing Institute last week, it was decided, at the instance of Mr. M. J. Sutton, that the committee should be allowed to afford nursing assistance to those in somewhat better circumstances who desired to make a small payment for the nurse at the time of illness, provided that such paid nursing do not in any way interfere with the nursing of the poor.—Sir Squire Bancroft, whose gifts to hospitals from his rendering of Charles Dickens's "Christmas Carol" exceed £10,000, will repeat the "reading" this winter in fulfilment of a limited number of promises, and with the same object.—The foundation-stone of a new cottage hospital, to be erected in Wellingborough, was laid the other day by the Marquis of Northampton. The site has been given by Mr. W. G. Woolston, a native of Wellingborough, and Miss Mary Woolston, who resides in the town, defrays the cost of the building, about £3,000.

### Lectures to Surgical Nurses.

By H. A. Latimer, M.D. (Dunelm), M.R.C.S. of Eng., L.S.A. of London, Consulting Surgeon, Swansea Hospital; Past President of the Swansea Medical Society; Lecturer and Examiner of the St. John Ambulance Association, &c.

### SPINAL DISEASES.—FUNCTION OF THE SPINAL CORD.—REFLEX ACTION.—POTTS' DISEASE.

I propose in to-day's lecture to deal with a subject which often presents itself to your notice, and in which your services are frequently called for: more often, indeed, than in the majority of illnesses. I allude to diseases of the spinal bones. In many of the cases we shall now be considering, the patients are reduced to such a condition of helplessness, or it is so essentially a part of their treatment that their movements shall be reduced to a minimum, that you are called upon to fulfil all the duties of your art; so I feel sure that in pointing out to you the especial features of spinal disease, and by explaining some of the symptoms which arise in them, I shall be both interesting and instructing to you.

I will begin by speaking of the anatomical peculiarities of the spine. It is made up, as you are possibly aware, of a number of bones. Twenty-four of these are distinct from each other; the first one, appropriately called the Atlas, supporting the skull and resting upon the one underneath it, and each successive one supporting the bone above it and resting on the bone underneath. These twenty-four vertebræ, or spinal bones, rest on a triangular bone at the bottom of the spinal column, which is formed by a fusion of five others, and this fused bone finally rests upon another fused bone, which forms a rudimentary tail, and has been much spoken of by Darwin and his followers as one proof of our descent from monkeys. Each vertebral bone consists of a body, which supports and rests on other bones; two side prolongations of bone, and two rounded arches which, meeting each other, coalesce and become prolonged to a spine. I purposely refrain from calling these various parts by anatomical names, for I do not want to do more than ask you to carry in your "mind's eye" their shape, and certainly do not require you to burden your memory with the acquisition of unnecessary terms. The facts which I wish to impress upon you are these: that the twenty-four true vertebræ, of which I have been speaking when I told you of some of these bones being distinct from each other, are so jointed together that they are allowed very free movements forwards, backwards, and sideways; that between each of them a layer of cartilage lies to act as a buffer in lessening shocks of concussion; that the arches of which I have been speaking enclose a space which is occupied by a mass of nerve matter, which is called the spinal cord; that this cord gives off a series of nerves, which escape at each side of each vertebral bone and so gain access to the body; that blood vessels for conveying that precious fluid run in grooves and holes of these bones, and are also protected from injury in their course; and that all the vertebræ are connected to each other by very strong ligaments. See, now, of what vast importance the health and integrity of each bone in this series is. Just as the strength of a chain depends upon its weakest link, so here, if one of these vertebræ becomes displaced by injury or disease, by pressing upon the spinal cord it interrupts or abolishes the flow of nerve force in all that part of it which lies below the seat of mischief; and such a result as this is so serious, for reasons which I now propose to give you, that spinal disease is justly regarded as one of the most serious ailments from which anyone can suffer.

What, then, is the function of the spinal cord? I will, in brief, tell you now. It contains a number of columns of nerve matter which serve as channels of conductions to and fro from the brain and body generally; and, in addition, it is the seat of special centres for the issuing of nerve power to organs in the body which must carry on their work without the knowledge or co-operation of the mind. These are the so-called reflex centres that you hear of from time to time. "Reflex action" is so interesting a phenomenon that I propose to turn aside from my main object for a few minutes in dealing very briefly with it. The way in which our organism is maintained at work is probably a matter to which few people give any consideration, unless their thoughts are purposely directed that way. In states of health the various functions of life are carried on almost without our knowledge, and the acts of respiration, of circulation of blood, of digestion of food, and of elimination of waste products, are proceeded with in order and regularity without the exercise of volition or will on our parts. That this should be so will be apparent at once when we consider the matter. It would never do for us to have to direct the working of those organs which aërate blood and circulate it, or which select nutritious materials from foods we have eaten, and that hurry out of our bodies the poisonous products of the wear and tear of our bodies. No! these complex acts must be performed in regular and orderly sequence if health is to be maintained, and they would manifestly not be so conducted were they only responsive to orders from the intelligent or conscious self. But these functions of organic life must have something to guide them and keep them going. This "something" exists, and is situated at various appropriate points in the spinal cord, at the so-called reflex centres. These centres, composed of collections of grey cells of nerve matter, may be compared to head offices which receive messages of wants elsewhere and then manage to send the help that is required. The medium of receipt of messages and transmission of orders is by a loop of nerves which link the organs concerned with its presiding centre in the spinal cord, and the whole process is carried on automatically. The centres are being continually stimulated by demands made upon them, and they send their orders back to the quarters demanding them without our cognisance; it is only when these messages are urgent and imperative, as the result of some disturbance, that we become aware of them at all. Take, for example, the act of breathing. Under ordinary circumstances this goes on in regular sequences of inspiration and expiration without our attention being directed to the matter. Owing to the fact that during its transit through the body the pure oxygen-laden arterial blood has given up this vivifying principle and has become changed into an impure carbonic acid-laden blood, the centre of respiration, which is situated in the higher part of the spinal cord, is stimulated to action; in response to this stimulation the centre transmits orders to the muscles which raise the chest walls and depress the diaphragm, and the lungs expand and become filled with pure air, from which the blood circulating in the blood vessels, in the walls of their air cells, takes a fresh supply of oxygen and gives out a load of carbonic acid gas. Should, however, these matters not proceed easily from any cause, and the breathing become hurried, we are apt to become aware of the fact, and we then supplement the work by voluntary efforts of our own in which the brain is concerned. From this example which I have adduced you will readily see how important it is that the special cord should be in perfect health.

### OUR CONVALESCENT FUND.

We have pleasure in acknowledging the sum of 10s. from Miss Katherine T. and 10s. from "A Nurse" for this fund.

### The Colonial Nursing Association.

### III.—ITS IMMEDIATE NEEDS.

It has been shown that, thanks to the efforts of Mrs. Francis Piggott and her friends, the Colonial Nursing Association is now in admirable working order. We may now fitly ask the question, What are its immediate needs? And, first of all, money is wanted. Not a large amount, but a sum which ought, because of its moderation and bearing in mind the object in view, to be obtained with ease. At the annual meeting at Stafford House last July Mr. Chamberlain moved the first resolution, which pledged the company to give their support to the executive committee in their earnest endeavour to raise the permanent funds of the association to £5,000. The committee had already received £1,200, and since then about as much again has been contributed. More than half the required amount has therefore still to be raised. If only the public will take the trouble to make themselves acquainted with the work the association is doing we are satisfied that they will find the means to relieve the hon. treasurer from any anxiety with respect to the actual sinews of war—war against diseases more formidable than any foes with whom England has to contend in the field of battle. The £5,000 is intended as capital for investment, on which 3 per cent., the highest rate possible, would give an income of £150 per annum. This, of course, would not suffice to defray the cost of carrying on. But the committee have every right to look for the further amount wanted from subscriptions. These at present represent £120 a year. With £240, which could be secured by existing supporters doubling their subscriptions, or, far better, by new subscribers, and £150 interest, the work of the association could, it is calculated, be efficiently done. That is to say, a net income of under £400 a year would pay the nurses' passage moneys, incidental expenses, printing, stationery, postages, messengers, assistant secretary's salary, and, in fact, all the expenditure of the association on the basis of existing operations.

As things are the association is under the double disadvantage of having to pay a portion of the expenses out of capital, while the capital itself is less than half the sum absolutely needed. This cannot too soon be remedied. The principle is bad, and, moreover, it is impossible to go on indefinitely paying expenses out of capital. At a meeting of the executive committee last week the question of a constitution was debated. In due time rules will be adopted and published. It may, however, be mentioned that the financial condition of membership is to be an annual subscription of 10s. or a donation of £5. There must be many persons sufficiently interested in the welfare of our Crown colonies and sufficiently well endowed with this world's goods to give a donation qualifying them as life members of the association. But there must be thousands who can afford, and are willing to give, an annual subscription of £10s. It is in this direction chiefly that the association should look for ordinary income. From the few the capital; from the many the small and numerous contributions which are so often the financial mainstay of an organisation.

The claims of the association to the liberal and systematic support of the public have already been pretty clearly indicated; but it is especially in regard to the West Indies and West Africa that help is required. Dominica, St. Vincent, and Grenada have all applied to the association, and, being small and poor colonies, they cannot at the outset employ nurses without supplementary financial assistance. It is but reasonable to expect that those in England whose interests lie in that part of the world, or who are aware what risks our countrymen residing on the shores of the most terrible coast of West Africa daily encounter, will respond to the appeal of the association. Mr. Chamberlain quoted, with telling effect, a letter from the officer in command of the West African Frontier Force on the Niger, in which he declared his belief that "the recovery of some of the young officers who had been struck down by that most deadly enemy of the white man—the black-water fever—was due to the attention and care of the nurses who had been sent out by the Colonial Nursing Association." Surely the mother country, out of the plenitude of her resources, will find money enough to fight "the most deadly enemy of the white man"!

There are other than financial needs, however, for which the association bespeaks the attention of the authorities, and of all who are concerned, to see that advantages which are enjoyed by the sick at home extend to those who have to be tended, either in their own homes or in the wards of hospitals, in Greater Britain. The time has, it is felt, fully come for the appointment of a commission of inquiry into the whole system of colonial hospitals. For example, in many places the hospitals are built on the worst possible sites, below fever level. Few of them contain paying wards, and there are no cottage hospitals. In very few is there any provision for quarters for nurses. It is obvious that no hospital ought to be erected on a site below fever level; that an efficient nursing staff should exist and be provided for in the estimates; that the establishment of paying wards for the benefit of the well-to-do classes in the colonies would be hailed with keen satisfaction; and that a certain proportion of cottage hospitals should be provided in up-country districts. The nomination of a commission, chiefly, but not entirely, composed of medical men, and supplemented in each colony where inquiries are held by the addition of two well-informed residents possessing indisputable local knowledge, is a step which may well be urged upon the Colonial Secretary. His action, so far, justifies the belief that little pressure will be necessary in order to induce him to sanction a step which would do much to hasten the revolution of nursing in some of our possessions. To quote Mr. Chamberlain's own words at the last annual meeting of the association: "Especially I attach importance to the recommendation which the committee have made, which they speak of as being an ideal—although, like most of their ideals, I think it is extremely practical—that is, that every hospital within our colonies or dependencies which is either supported by the society or by Government funds should have one or more of these nurses attached to it, who should devote themselves, where circumstances require it, to the service of private patients, and should give the rest of their time to the work of the hospital, which should become their head-quarters. That is an ideal which I hope we may realise before very long, and, as I have said, the results of the experiment are thoroughly satisfactory."

How greatly circumstances call for the revolution may be demonstrated by the citation of a single fact. It will hardly be credited by the uninformed that French sisters are responsible for the nursing in a number of our colonial hospitals. At Colombo, the capital of Ceylon, French sisters are in charge of the Government Hospital, as they are also at Aden, Singapore, Mauritius, and many other places. In the opinion of the executive of the Colonial Nursing Association, an opinion which will be widely endorsed outside, a useful and illuminating purpose would be served by the issue of a return showing in how many of our hospitals French sisters have excellent posts, and why it is considered desirable to employ them. Without casting the slightest reflection on their character or capacity, it may be profitably inquired why the services of English nurses are not utilised in English hospitals built and maintained by English money, and whether it tends to the efficiency of the care of our sick if their nurses are prevented by religious vows from remaining in the room in the event of delicate operations, leaving their patients to the care of native dressers! The Colonial Nursing Association, at any rate, does wisely and well not to be concerned only about the supply of the wherewithal for its own necessities.

### The Nurses of St. George's Hospital.

### A CHAT WITH THE MATRON.

By Our Commissioner.

The workmen are still in possession of St. George's Hospital. They were fully in evidence when I called the other afternoon to see the Matron. They commenced in 1897; it will certainly be far into 1900 before they have finished. The hospital has never been closed owing to alterations, but everyone in it will be glad when the inevitable noise, dust, and confusion finally cease.

Miss Smedley was kind enough to enable me, by personal inspection, to form some idea of the nature of the improvements. All the kitchens will be removed from the basement to the top of the building; the accommodation for nurses will be improved. But the nurses are already well provided for. Their spacious sitting-room, which was opened last Christmas, commands a magnificent view of Hyde Park. There is a dressing-room specially set apart for those who sleep at the home, with lockers for each.

"When the alterations are completed," said the Matron, as we chatted in her office, "there will be a few extra bedrooms for nurses. It is very difficult to build in such a public place as this, and also because of the height. Moreover, we have almost sufficient accommodation for the present staff. But it is a great pleasure to have the upstairs quarters improved, and an absolute luxury for every nurse to have her own bedroom instead of a cubicle."

"Will the staff be increased?"

"Possibly. The number is 130, of whom 22 are sisters. The remainder are staff nurses and probationers. The majority, or nearly 80, live in the home in Montpelier Street."

"Do they include any of the probationers?"

"Some of the seniors. The night nurses and the day staff live at the home, and all the junior probationers here. There is no private nursing staff in connection with the hospital."

"How long has the home been opened?"

"Five years. It contains a dining-room, sitting-room, and writing room, as well as a bedroom for every nurse. There is a fireplace in each, and the electric light is in use throughout the building. The quarters here are also very comfortable, with the advantages of fresh air and plenty of exercise."

"Which do the nurses prefer—the home or the hospital?"

"It is a question of individual taste. Probationers always come first to the hospital, and later on they go to the home. Of course it would be much more convenient to have the home close to the hospital, but the Governors could not get either the ground or the houses any nearer."

"Perhaps you would tell me the minimum off duty?"

"Two hours a day. In the second and third years the probationers, instead of going from six to eight in the evening, have from six to nine, or six to ten, according to seniority. The day nurses are allowed a day off each month."

"And the night nurses?"

"They are permitted to stop out of the hospital one night each month. This is an arrangement that has been very much appreciated. The nurses largely avail themselves of it, especially in the summer."

"When did it come into operation?"

"Last spring. As to holidays, all the nurses get a fortnight, and those who have to travel a long distance are given an extra day. The sisters have a month's holiday and a day off each month."

"I think that this year you have inaugurated new regulations as to lectures?"

"Yes, they deal with the whole question of education. Under these regulations nurses in the first year of training are taught the elements of practical nursing by the sisters in the course of the ordinary work, and by class demonstrations given under the superintendence of the matron. They are required to pass an examination in elementary nursing during the first year's training. It is wholly practical, and until it has been passed probationers will not be permitted to proceed to the training arranged for the second year."

"And the other years?"

"During the second and third years of training nurses who have satisfactorily passed the first examination, in addition to receiving personal instruction from the sisters, will be required to attend classes and demonstrations dealing with more advanced subjects of nursing. They will also have to attend lectures on medical and surgical nursing given by members of the staff. At the end of the three years' training those who have passed the first examination and have attended the classes and lectures will be examined as to their nursing capacity and knowledge. Nurses who in the examination reach the required standard will be placed in Class I., and the remainder, provided they reach the qualifying standard, in Class II."

"What happens in respect to a nurse whose reports are not satisfactory."

"The Nursing Committee reserve to themselves the right to discharge her at any period, or of refusing her admission to the next examination."

"Have you a great number of applications for admission as probationers?"

"I always see candidates before a formal application is made. They come on trial for a period varying from one to three months, and appointments are made every quarter."

"Do you have to reject many after serving the preliminary term?"

"On the whole few fail. But it is a very good thing to have a sifting time."

"Are your general rules of this year's date also!"

"Yes; but they were drafted to a large extent on the basis of the old ones. The policy throughout has been to make changes by degrees—to 'hasten slowly.'"

"I conclude that the probationers must continue in the service of the hospital for four years?"

"Each of them, having been subjected to a preliminary term of trial, and recommended by the matron to the Nursing Committee, has to sign an agreement to remain for four years, dating from the day of admission. The rate of wages is £10 for the first year, £14 for the second, £22 for the third, and £26 on passing the qualifying examination for a staff nurse."

"What about uniform?"

"Nurses are provided in part with indoor uniform, and they are required to wear outdoor uniform, concerning which they receive instructions from me. The outdoor uniform is worn, except on the monthly or annual holiday, or except by special permission."

"Is any special provision made for the amusement or recreation of the nurses?"

"I have a strong feeling that nurses should amuse themselves as they like. People are very kind in sending us tickets for theatres and concerts. We have concerts twice during the winter given by the staff of the hospital, to which each nurse can invite a friend. Last season one of the surgeons gave a very successful theatrical entertainment to the nurses."

"Have any of your staff been selected for service in South Africa?"

"None of them are on the first reserve. But many are very anxious to go. I think that the desire to be sent to nurse the sick and wounded is very general."

### The Dangers of Nursing Typhoid Fever.

HOWEVER confident we may feel that typhoid fever is not directly infectious from case to case, the fact remains that those who have to deal with patients suffering from this disease are attacked by it in undue degree. This is strongly borne in upon us by our having to record in one week three deaths in our ranks from this one cause, for although it is just possible that in each of these cases the malady may have been contracted in a manner unconnected with the nurse's calling, there seems every reason to believe that the infection was in some way contracted while on duty.

Why is it that nurses suffer so from this disease? Sanitarians have for so long drummed it into the ears of all the world that typhoid fever is a filth disease conveyed by water, and the doctrine of the non-infectious nature of the disease, so far as personal contagion is concerned, has been so strongly insisted upon, that it is to be feared that some degree of carelessness has grown up in regard to those details in the management of such cases which must be religiously observed if the danger of infection is to be avoided.

It is true that the infection of typhoid fever goes in at the mouth—as probably do the infections of most diseases—but it is not true, as is so often thought to be the case, that it is by means of water or of milk that it is mostly carried there. So far as epidemics are concerned, no doubt water and milk are the most common vehicles by which the disease is spread; and so far as epidemics are concerned nurses take their chance with the rest of the community. But the typhoid from which nurses especially suffer is that caught in the sick-room, and the principal modes in which it is so caught are three—by dust in the air, by dirt on the fingers, and by food in the pocket. If a nurse is utterly careless, there are plenty of modes by which infection may gain access, but in the majority of cases it is caught in one or other of the above-mentioned ways.

The great thing to remember is that the infective material is contained in the excreta of the patient, probably in all the excreta, but more especially in the fæces and the urine. This is the prime fact upon which all the rest hinges. The next fact is that in attending to a helpless patient it is impossible for the nurse to avoid to some extent soiling her hands with these infective matters. Some nurses manage better than others, but no nurse can properly do her work without exposing herself to this risk. This is probably the chief cause of danger, and one of the main reasons why young nurses, and those whose experience in fever nursing is not large, suffer more than others do from typhoid. The risk must be recognised and means must be taken to reduce it to a minimum. The hands must be washed, and perhaps disinfected, every time that they have run even the remotest chance of becoming soiled; the nails must be kept short; the nail brush must be freely used; and certainly before food is partaken of the hands should be thoroughly disinfected in perchloride of mercury solution, 1 in 1,000.

But what we wish to insist upon is that safety is not to be attained by this formal disinfection of the hands merely before eating, if at other times they are allowed to remain soiled. Few people have any idea how often the hand is carried to the mouth during the course of the day. It is a bad habit, and one to the conquering of which nurses should especially train themselves, for it is a habit which vastly increases their liability to catch infection from those they may be nursing. In any case, however, when nursing typhoid the nurse's hands should be washed many times a day, besides the necessary disinfections after attending to the patient. The next habit which we have to speak of is that of staving off the pangs of hunger—and they really are pangs sometimes—by the occasional nibbling of biscuits kept in the pocket for the purpose. The long hours of work, the responsible nature of the work, and the fact that the appetite is often considerably interfered with by the sort of duties the nurse in such cases has to perform, all tend to set up a sense of "craving," a feeling of emptiness, just that feeling which led the Gamps of old to fly to gin and water. Of the reality of the distress there can be no doubt whatever. It may so happen that just before a meal is due something may have to be done which may entirely take away the appetite for the time. Practically that meal is lost, and in an hour's time the nurse is thoroughly empty and wretched. In a hospital the discipline, and in private work the fact that she is in sole charge and cannot get off, render it impossible for her in such circumstances to obtain any proper food for perhaps three or four weary hours. The alternative is biscuits, which is a most dangerous adventure.

There is always something more or less surreptitious about biscuits. No one ever openly sets to work to eat a meal of biscuits; they are kept in the pocket; they are nibbled one, or, indeed, only half a one at a time; they are taken "in between" snatches of work; and when that work is the nursing of a typhoid case they lead directly to infection. We do not say that the well-trained nurse will do such things, but the craving of an empty stomach is hard to bear. The next danger that we have to speak of is dust, and this is a danger from which even the best trained nurse cannot entirely escape, especially when nursing in small houses which are poorly supplied with the clean linen which these cases so urgently require. Whenever any soiling of the linen takes place, whether by fæces, or urine, or, for aught we know, by expectoration, if the stain be allowed to dry an infectious dust is liable to arise when the linen is handled. And some amount of soiling is very difficult to avoid; and some amount of drying it is almost impossible to prevent, when the supply of linen is limited. But to be forewarned is to be forearmed. Every care should be taken to prevent soiling of the linen; when soiled it should never be dried, but should be placed at once in the disinfectant solution. All needless shaking of linen should be avoided, and, lastly, the nurse should cultivate the healthy habit of breathing through the nose and keeping the mouth shut while doing anything about her patient.

### The Improvements at the Royal Orthopædic Hospital.

THE Earl and Countess of Denbigh were "At Home" at the Royal Orthopædic Hospital, 15, Hanover Square, on Monday. Lord Denbigh was elected president in June, and took the opportunity afforded this week of making the acquaintance of the governors and various friends of the hospital. The building was closed in the summer for three months in order that the sanitary recommendations of Professor Corfield and the structural alterations under Messrs. Young and Hale, architects, might be carried out. Consequently, everything was fresh and bright. The wards were decorated with palms and plants. Amongst the invited guests were: Mr. H. H. Marks, M.P., Mr. Ernest Flower, M.P., Mr. and Mrs. Bernard Parkes, the Rev. R. H. Sinclair, Professor Corfield, Dr. and Mrs. Bulleid, Mr. and Mrs. Bernard Brodhurst, Mr. H. A. Reeves, Mr. Charles Re[illegible]d, Mr. Henry F. Baker, and others. General satisfaction was expressed concerning the improvements. The hospital is the [illegible]est in the country for the treatment of deformity, and was founded sixty years ago. It has treated 81,000 patients during that time, and is now inadequate to supply the needs of the patients needing help. The committee are accordingly appealing for the sum of £2,000 to defray the expenses recently incurred.

### Some Wardmaids I Have Known.

By E. M. Fox.

The good ones are few and far between, and they seldom stay. Many years' experience of them has caused me to regard them with a somewhat pessimistic eye, and makes me expect less and less from them as time goes on. Even from the much-abused domestic of suburban notoriety one expects more, for she lacks somehow the airy independence of the dweller in hospitals.

My first impression of them was not favourable. I was a very new probationer at the time, for it was, in fact, my first day in the ward. Spotless in the perfection of my new clean uniform, and filled with satisfaction at the whiteness of my collar, as yet unsullied by London laundresses, I was washing up lunch things in the ward kitchen, and that maid was just behind me. Presently something happened. I felt a dull, soft thud on my back, and a sense of cold moisture oozing down my neck. I gave a slight shriek, and the maid echoed that shriek, ending with an hysterical giggle. In reaching for a shelf above me her foot had slipped, and she had upset all the remains of yesterday's cold rice pudding down my back. It felt damp and nasty, and "Ichabod" was written legibly on the back of my nice new dress. I thought a great deal, and considered it much to my credit that I did not say things. She laughed too much to apologise adequately, I fancied, and somehow after that I did not take kindly to wardmaids.

There were a great many of them at our training school, and most of them considered themselves far and away superior to us mere probationers. They lorded it over us in the ward kitchen, and refused to let us use the saucepans and dishes we wanted. They complained to Sister that we let milk boil over their stoves after they had cleaned them, and that we took their dusters and hid their tea-cloths. That was not true, for it used to be they who took our dusters, but we could never prove the case against them, and the scoldings fell to our lot instead of theirs.

They fetched letters and parcels in those days for the wards, and we would see a little knot of them assembled at the foot of the stairs discussing and criticising the handwriting on the letters addressed to us, or even unblushingly reading our post cards!

There was one wardmaid I positively feared in those now almost prehistoric times when I was yet a stranger in the land. She was big and tall, with great, bold, black eyes, a heavy step, and a loud voice. She was fond of discussing the salaries of the nurses, and of asking us on quarter-days if we had had our pay, and how much we got. If one of us went to the matron's office she was always very anxious to know why we were sent for, and what we had been doing. She did not hesitate to report all our delinquencies, real and imaginary, to Sister, and she expected us to be perfection, though she did her own work very badly indeed. I think Sister herself was a trifle afraid of the big, overbearing creature, and I know none of us were at all sorry when, at last, she left, "to better herself," she said.

After her I can carry my memory back to a long train of more or less incapable, stupid, or otherwise impossible girls, with, alas! very few exceptions. One there was, certainly, who stands out brilliantly from among her fellows—a clean, hardworking, pleasant-looking, little Welsh girl, whose kitchen it was a pride and a delight to go into at all hours. Her brasses were always bright, her tins shone like silver, and the fame of her was spread abroad throughout all the hospital. She never forgot to make up the ward fires at the right time, and one never had to remind her to move the lockers when she was sweeping. But such perfection was bound to be evanescent. There came a day when Rose thought she would prefer private service, and deserted us, much to our grief; and straightway the saucepan lids lost their polish, and the door handles knew her determined touch no more. She was gone, and any number of regrets would not bring her back.

She was followed by another list of feckless ones, who broke the crockery, destroyed the furniture, and spoiled the beef tea. One particularly helpless young person followed Sister on her morning round with the house-surgeon, holding a limp chicken in her hand, and entreating to be told what she must do with it. "Why, cook it to be sure," Sister answered, with some asperity it must be confessed; but the damsel replied, even with tears, that that was just what she could not do. She did not know how, and had never been used to such things! In the end Sister had to cook that chicken herself, while the maid stood agape wondering at the things which were done.

Yet another wardmaid I remember, who on her arrival at the hospital cried for nearly three consecutive days, and finally went home, after spending much time weeping just outside her ward, sitting on the linen basket, the observed of all observers.

Another came one Friday evening, and by Sunday afternoon, having decided that the hospital was no place for her, quietly went off duty, as was usual, and returned no more, evidently deeming the customary month's notice as an obsolete usage, unworthy the attention of an up-to-date domestic. Her successor was a young lady, who, on being sent to tidy the Sister's bed-room, only succeeded in simply making hay in it, and frankly admitted, without the least reserve, that she had never made a bed in her life. This girl was nineteen, and had probably been destined by her mother for domestic service of some kind all along. One cannot but marvel at the short-sighted policy of some of these girls' parents, who send their children out into the world to earn their living totally unequipped for the battle of life. So these ignorant and careless ones greatly add to the anxieties of the already much-tried sisters of wards, who can trust them to do nothing properly unless under their own immediate supervision, and who naturally need most of their teaching and training powers for their probationers.

Good-natured and willing enough some of them are, and certainly not lacking either in ingenuity, if one may judge from the device of a new wardmaid who, not being able to find a suitable tin in which to cook the chicken, baked it on a small tea-tray, and, flushed and triumphant, brought it to show Sister, expecting commendation for the deed. The same girl also on another occasion wanted to be allowed to boil some greens in the ward steriliser, but was fortunately stopped in time. She was ever good-humoured and cheerful, singing exasperatingly over her work at a time when grave and august surgeons were visiting the ward. She broke innumerable things, including on one occasion all the contents of Sister's tea-tray, save a small tea-cup and a cracked sugar basin, which naturally escaped the general smash-up, not being good for much. "I'm awfully sorry," was her one unfailing comment on exploits of this kind, and the remark being made generally with the widest of smiles on a face that was round to begin with, was far more irritating to one's nerves and temper than "I don't care" would have been.

In recounting the various characteristics of the wardmaids it has been my lot to meet, I must say in justice to them that whatever faults they may possess—and their name is legion—disloyalty to their ward or nursing staff is not to be reckoned among them. No matter how badly they do their work, nor how many scoldings they receive, they never fail when off duty to extol "my ward," "my sister," "my staff nurse" as being perfect, to the obvious detriment of every other ward, sister, and nurse in the hospital not honoured by their attachment.

Hot arguments, not seldom widening into downright quarrels which have been hard to quell, have been known to occur in the dormitories anent the rival merits of different wards, quarrels which have ended in the most bosom friends becoming sworn foes and refusing to speak to each other any more. Intensely human these girls are, with all the faults of their age and class, and yet, tiresome as they can be, they help to lend life and colour to the hospital, and are sorely missed when they leave suddenly and their place has to be filled by those who do not know the ins and outs of that particular ward. Their mistakes may be annoying, their carelessness irritating, but we who live among them and are dependent in a large measure upon them are fain to confess that in spite of all her defects the genus wardmaid is a necessity, for we do not want to return to the pioneer days of nursing, when nurses and probationers did all her work and the wardmaid was unknown. So nothing remains but to make the best of them, to try and improve them, and to endeavour to believe they are not so bad after all.

### Papers for Probationers.

By a Sister.

### TAKING THE PULSE.

We have seen the importance of taking temperature intelligently, and the same care must be exercised with regard to the pulse. It is not sufficient just to count a patient's pulse and mark it down on a chart. To be a good nurse you must know much more about it than that. Though a probationer is not expected to understand the varieties of pulses and the conditions and diseases they denote, she is expected to learn, both for her own and her patients' sakes. Let me warn you against a very common fault which young nurses fall into, and that is, ignoring the pulse altogether, and thinking that the temperature is the one thing needful to obesrve. In many very dangerous conditions the temperature remains normal or thereabouts (notably in many forms of heart disease). I have even nursed a case of pneumonia—a most unusual one, I grant—where the temperature remained between 97 deg. and 99·4 deg. all through the ten days the patient was in hospital, and yet he died—and of pneumonia. An ignorant and unobservant nurse might have made that common mistake of thinking, "Oh, he's all right; he hasn't got a temperature." A doctor can tell how the heart is doing its work by means of the stethoscope, as well as by observing the pulse, but the nurse's only guide, apart from symptoms, is the pulse. The pulse is the rhythmic rise and fall of the arterial walls as they expand to receive the blood pumped into them by the heart, and contract between each impulse as the pressure lessens, forcing the blood-stream onwards. Of course the pulse can be felt in every artery, but the most accessible points are: (1) Over the radial artery at the wrist, about an inch and a half below the junction of the thumb, in a line with the inner side of the first finger; (2) over the carotid artery, below the angle of the jaw; (3) over the temporal artery, just below where the top of the ear joins the head; (4) over the posterior tibial behind the inner ankle bone. This last is a useful spot with sleeping children, but the first point at the wrist is the most generally convenient. Every pulse ought really to be taken for a full minute, but in a hospital this is well-nigh impossible owing to lack of time. Still, every pulse taken for the first time should be counted for a full minute, as should all irregular pulses and those taken every four hours.

The principal points for a nurse to observe with regard to the pulse are: (1), Rate; (2), rhythm; (3), character; (4), relation to temperature and respiration.

Rate.—The rate or rapidity, apart from disease, is increased when a patient is standing, if he is excited, or has just undergone extra exertion, such as a fit of coughing, &c., and should therefore not be taken under those conditions. The normal pulse rate of an adult is about 72, but varies between 50 and 80. In some diseases or conditions the pulse is slower than normal, but in most cases it is increased, especially in fever and in most inflammatory conditions. A rapid pulse indicates weakness of the heart; the actual heart beat is the same length, but the time of rest in between each is encroached on, and the heart's strength becomes exhausted. Stimulants and heart tonics are given to steady and diminish the rapidity of its action. Where the pulse is extremely rapid, each beat, as it were, being merged into the next, it is called a "running pulse." In most cerebral diseases and in some heart cases the pulse is abnormally slow. Some times an abnormally slow pulse is due to the fact that all the heart beats are not sufficiently strong for the pulse wave to reach the distant arteries, so that while the heart beats 80 times per minute only 40 expansions can be felt at the wrist.

Rhythm.—In health the pulse beats are usually quite regular, in disease variations occur. The beats may be regular as to time but drop a beat occasionally, perhaps one in every four or five, or at longer and irregular intervals; the pulse is then said to intermit or to be intermittent. The beats may be coupled or occur in threes or fours, alternate beats may be strong or weak, a "pulsus alternans." Again, there may be no sort of order in the beats, either as to strength or rhythm, it is then described as an "irregular" pulse. Another peculiarity is "dicrotism." A pulse is called "dicrotic" when each beat is followed, before the first has died away, by a second very soft wave like an echo or reflection, as it were.

Character.—The pulse may be hard and incompressible or soft and compressible, that is to say, requiring some force to arrest it, or being easily stopped by slight pressure. The hard or high tension pulse is due to the strength of the heart beats, the quantity of blood pumped into the arteries by the heart, and the pressure exercised by the blood vessels on their contents. A soft or low tension pulse is produced by a feebly acting heart, a smaller blood supply to the arteries, due to leakage at the valves of the heart, and dilatation of the blood vessels, thus allowing little resistance to the passage of the blood. An apparently hard pulse may have nothing to do with the blood pressure, but be due to a thickened condition of the arterial walls which prevents their expansion.

Relation to Temperature and Respiration.—The pulse rate is usually increased with a rise of temperature somewhere about four beats to a degree. In typhoid or enteric fever the pulse is slow in proportion to temperature, in peritonitis it is much increased. These are only two instances, but there are many more, and you should make a habit of observing the relations and its variations.

In the first instance the pulse should be taken in both wrists, and you should notice if the beats in both wrists are equal in strength and occur simultaneously, or if one is later than the other "delayed" pulse. Inequality in these respects is an important symptom, pointing to pressure, frequently due to an aneurysm. Your aim then in taking a pulse should be to ascertain not only its rate, but whether it is regular, irregular, or intermittent, hard or soft, strong or feeble, and what relation it bears to the temperature.

### To Nurses.

In order to increase and vary the interest in the *Mirror*, we invite contributions from any of our readers in the form of either an article, a paragraph, or information, and will pay a minimum of 5s. for each contribution. All rejected manuscripts are returned in due course, and all payments for manuscripts used are made at the beginning of each quarter, *i.e.*, January 1st, April 1st, July 1st, and October 1st.

### Echoes from the Outside World.

### AN OPEN LETTER TO A HOSPITAL NURSE.

Up to the time of writing on Wednesday evening the lull in the war has continued. The old adage of "no news" being "good news" seems, in this instance, to be true, for as long as Ladysmith holds out, only an occasional message, sent by heliograph, pigeon post, or native runner, is likely to reach us. One dispatch by hand came on Tuesday. It told us that the inhabitants of the beleaguered town were still cheery and full of confidence, though bread was selling at three shillings a loaf. Think what that means to slender purses! The soldiers must rejoice that they do not have to buy their own rations. Two stories are current which need to be received with caution until they are fully confirmed. The more important refers to the anxiety of President Kruger to get back the man, Nathan Marks, who came into Ladysmith to spy, disguised as an ambulance driver. As yet the summary justice which is usually meted out to a detected spy has not been administered to him, but the President is reported to have threatened that unless the captive is at once released he will shoot six of the British officers who are now his prisoners. It is difficult, considering the number of Boers we hold ourselves, to believe that he would dare to do so, but I repeat the rumour for what it is worth. Then there is another statement said to have been made by the Chaplain to the Irish Fusiliers, that the reason the men and the battery were captured was because a subordinate officer, supposing that all but the ten men whom he had with him had been slain or dispersed, hoisted the white flag in error, and so all had to surrender. There are two pleasanter items of intelligence. One is the appointment of Sir Charles Warren to the command of the Fifth Infantry Division now being mobilized for service. As General Warren was most successful in his conduct of the Bechuanaland Expedition in 1884-85, he should be the right man in the right place. And, in order to show their sympathy in a practical form, the Danish Government is going to send out to our troops a present of 50,000 boxes filled with butter. As butter is not always easy to get in South Africa, and as, moreover, Danish butter is very good, I expect our soldiers will say "Three cheers for the Danes" when they come to sample. From Mafeking an undated message stated that "every one in the town is most cheerful, and determined to resist the attacks to the utmost." There is said to be no scarcity of provisions or water, but everyone is reported to be very tired of "dodging shells."

---

I take the following from private letters from Natal this week: I wish we could tell you how we all—I mean the whole colony of Natal—appreciate the stand England has taken this time, and the sacrifice she is making to secure peace for us eventually. We are aware that the war will be no child's play for Natal, but now that we know England, the Empire, is behind us in full force, we can fight with very different feelings. Already, of course, distress is all around, though the worst sorrow, of relatives taken from us, has yet to be experienced. For some months the richer men in Johannesburg have been reducing their staff, or leaving the country, so that want has been more or less rife, and money has been scarce. Then, when the final rush came, men, women, and children had to come down to Durban and Cape Town in open trucks, often fifty hours at a stretch, without food or shelter, so that those who were delicate will feel the effects all their life. Added to the discomfort was the knowledge that tons and tons of luggage had been left behind, all to be looted by the Boers, so that many lost everything that they possessed. Now, with the mines closed, the banks all robbed by the Transvaal Government, no dividends coming in, no salaries, no rents, no income of any sort, can you picture the state of families who in January last were wealthy? Of workmen with good wages, who had saved to buy a house and furniture, adrift with no work, no house, no furniture? So you see we have need of all you are so generously subscribing to the Transvaal Refugee Fund at the Mansion House.

---

Here is another extract: Letters from the Natal volunteers at the front are written in a cheerful strain, which goes some way towards keeping our spirits up, but, of course, it is a trying time for our boys, many of whom are young and not used to roughing it. There is a good deal of dysentery, owing to the bad food, or rather the bad cooking, which renders it unpalatable—one fellow says he would not recommend the camp cook even to his worst enemy—and the want of sleep is a good deal felt. Never more than four hours at a time, and never twice in the same place, for fear of being surprised by the enemy. We are busy making little waterproof bags, in which we are going to put home-made Boer rusks, sticks of chocolate (chocolate is splendidly sustaining), tablets of "Maggi soup," and a couple of boxes of matches. These we shall send up to our special friends for distribution amongst the others in the corps. In the last letter which we had from Harrismith—there will be no more, I fear, till the war is over—we learnt that the English were fearfully anxious, as they had no news except what the Boers told them, and that was all of victory, with fearful slaughter and defeat of the British. The nurse who had been officially nominated head nurse of the Harrismith hospital complained bitterly that she could not even get the necessaries for her patients, and she found it difficult to work under the Hollander, a total stranger who had been appointed doctor instead of Dr. Wilson.

---

I have been to see "The Black Tulip" at the Haymarket. A good many of the critics of the dailies saw fit to sneer at the play, because it has no desperate situations, no unpleasant suggestions, no doubtful moral. But there was abundant evidence on Saturday that there is still a large proportion of the public who enjoy a thoroughly wholesome play, for the house was packed from roof to floor, and many who would have liked to stay were turned away for want of room. The plot may not be of a highly exciting character, but the interest never flags, and Miss Winifred Emery is almost as winsome as she was in "The Little Minister." Higher praise could not be given, and it is easy to understand that she spoke the truth when she said that there were dozens of young men always ready to pay her attention. Surely, never did a gaoler's daughter learn to read under more trying circumstances! The poor tulip-lover, imprisoned within a cell of the most limited dimensions, is only just able to get his hands sufficiently far through the bars of the grating to point to the letters. Here each day after dinner, whilst the gaoler sleeps, comes his daughter Rosa, and, holding the book up to the grating, receives instruction in the art of reading—and, need I also say, in the art of loving? —from the prisoner within. It makes a most pretty picture, and Cyril Maude and his wife always play charmingly together. Gryphus, the gaoler, is a most repulsive person, and his laugh, which his victim mistakes for a groan, because it is so lugubrious, is very cleverly done. The scene at the end in the Gardens of the Horticultural Society at Haarlem, when William of Orange shows himself to be a second Solomon, is very inspiriting, and the appearance of Miss Emery in the national wedding dress of the period is so bewitching that prospective brides who saw her on Saturday afternoon must almost have wished themselves Dutch girls, living in the seventeenth century, that they, too, might wear such a quaint, delightful gown.

### Bristol General Hospital.

Nurses' Prizes.

The medals and prizes gained by nurses at the Bristol General Hospital were distributed the other evening by Mr. J. Storrs Fry, chairman. Mr. Fry subsequently entertained the nurses at an "At home," to which also many members of the committee were invited. The medals were given for the first time. The following is a list of awards: Gold medal, Nurse Wason; silver medal, Nurse Bottomley; certificates of merit, Nurse Friend, Nurse Parish, Nurse Allbutt, and Nurse Castle. Physiology, second year nurses —First prize, Nurse Bale; second prize, Nurse Richards; first year nurses, first prize, Nurse Bridges; second prize, Nurse Page. Anatomy, second year nurses—First prize, Nurse Richards; second prize, Nurse Pictor; first year nurses, first prize, Nurse Page; second prize, Nurse Bridges. Medical and Surgical Nursing, second year nurses—First prize, Nurse Bale; second prize, Nurse Edwards; (first year nurses, first prize, Nurse Bridges; second prize, Nurse Page; first prize, Nurse Hamsteed; second prize, Nurse Talbot.

### Aberdeen Royal Infirmary.

An improvement has recently been made in the Aberdeen Royal Infirmary in the form of a subterranean passage leading from the Nurses' Home to the surgical wards. This enables the nurses to cross to and fro on wet days without the risk of cold or damp feet as formerly, and will be of great benefit to all the nursing staff during the winter. A similar passage leads from the new medical pavilion to the basement, where the operating theatres are situated, and thus connects the two.

### Appointments.

The Institute for Trained Nurses, Melville Street, Edinburgh.—Miss Milne has been appointed Matron. She was trained at the Edinburgh Royal Infirmary. She then became matron of the hospital at Rosario, in the Argentine Republic, and afterwards matron of the British Hospital at Monte Video. Returning to Scotland, she was appointed night matron at the City Hospital, Edinburgh, and subsequently night superintendent of the Western Infirmary, Glasgow.

Bristol General Hospital.—Miss Mattick has been appointed Night Superintendent. She was trained at the Bristol General Hospital, and for the past four years has been sister in the children's and female surgical wards.

### Minor Appointments.

Hunslet Union Infirmary.—Miss Ada Fanny Lockett has been appointed Superintendent Nurse. She was trained at the Leeds General Infirmary, and Queen Charlotte's Lying-in Hospital, London, and has since held the positions of assistant matron and midwife at the British Lying-in Hospital, London; home sister at the Nursing Home, 5, Albion Street, Hull; and superintendent nurse to the Huddersfield Union.—Miss Elizabeth Emma Lownds has been appointed Charge Nurse. She was trained at Queen Charlotte's Lying-in Hospital, and the Royal Infirmary, Hull, and has since been engaged in private nursing.

Fulham Infirmary.—Miss Rose Wells Pascoe has been appointed Superintendent of Night Nurses. She was trained at King's College Hospital, London, for three years; for three months at Queen Charlotte's Hospital for Midwifery; and in massage at the Elgin Nursing Institute. She has only just finished training.

### Presentations.

Royal London Ophthalmic Hospital—Miss Robinson's Coming Marriage.—Few matrons have more entirely identified themselves with their hospitals than has Miss Ada Robinson with "Moorfields," over the nursing department of which she has reigned with such conspicuous success for some years. Her departure this week leaves a blank which it will take long to fill, though it is a satisfaction to those with whom she has worked so harmoniously that her connexion with the hospital will even now not be wholly broken, as in a short time she is to marry Mr. Bland, who as secretary to the hospital has done so much to forward its interests. Mr. Bland and Miss Robinson will be the recipients of very many good wishes from their numerous friends in the hospital world. Before the departure of Miss Robinson, the sisters and nurses presented her with a charming travelling clock, inscribed with her name, date, &c., in memory of the pleasant relationship always existing between the nursing staff and their Matron. Miss Robinson has seen the hospital fairly started in its splendid new quarters in the City Road, and it is not surprising that the heavy work of the past months, into which she has put so large a measure of enthusiasm, has made it necessary for her to give up her post earlier than had been anticipated, to the great regret of the committee and staff. The committee are to be congratulated on their choice of a successor to Miss Robinson. Miss Richards, who takes up her new work at once, has been one of the most popular of sisters at the London Hospital.

Liverpool Stanley Hospital.—On Thursday evening last week an exceedingly interesting event took place at the Liverpool Stanley Hospital, when the matron, Miss A. A. Lewis, who has completed twenty-one years' connection with the hospital, was the recipient of a handsome illuminated address, accompanied with a purse of gold, by the committee, staff, and her colleagues. The address, which was signed by all the subscribers, referred in eloquent terms to her devotion to her duties, and testified to the affection in which she is held by all connected with the hospital. The presentation was made, in the unavoidable absence of the president of the hospital, the Rev. Canon Lester, by the chairman of the House Committee, Mr. Thomas Backhouse, who delivered a happy speech. This was followed by a most enjoyable concert, organised by the hon. secretary, Mr. J. G. Bennett, who occupied the chair. The presentation and concert took place in the out-patient department of the hospital, which was gaily decorated for the occasion.

Western Infirmary, Glasgow.—At a gathering of nurses in the drawing-room of the Nurses' Home, Western Infirmary, Glasgow, on Saturday evening, Miss Milne, night superintendent, was presented with a handsome silver Queen Anne tea service, bearing the inscription, "From the Nursing Staff," and half-a-dozen silver teaspoons with her initial. In addition to these gifts from the nurses she was presented with a couple of silver jelly spoons, and case containing a pair of silver butter knives with mother-of-pearl handles, from the maids. Miss Milne is leaving, after five and a half years' service, to assume the position of Matron of the Institute for Trained Nurses, Melville Street, Edinburgh. Dr. Macintosh, superintendent, in making the presentation, expressed the regrets of all at Miss Milne's departure, and their hearty good wishes for her happiness and success in her new undertaking.

Bristol General Hospital.—Miss Annie Ellis, who has left the General Hospital, Bristol, where she has acted as night superintendent for five years, in order to take up her work at the new Bristol Convalescent Home, which was opened by the Queen on Wednesday, was presented the other evening, by the nursing staff of the hospital, with a silver tea service, pictures, and silver spoons, from the matron, assistant matron, and secretary.

### A Book and its Story.

MISS HENRIETTA FOWLER'S NEW ROMANCE.

In her charming and unique story, "A Corner of the West,"* Miss Henrietta Fowler contributes a pleasant addition to the wholesome school of social romance pure and simple—a school, unfortunately, too little represented in these waning days of the nineteenth century. No unpleasant complications, no doubtful situations, mar the pages of this delightful story, which is pervaded throughout by an old-world atmosphere of calm stillness and out-of-date serenity. In the setting of the homelier scenes there is a vividness and alertness in the movement of the more interesting characters, with glimpses of the outer world in Petronel Merrivale's home, and the smart house parties from time to time gathered there. Petronel Merrivale and Alison Royse form a study in contrasts. They are girls of the "new" type, each fulfilling herself in different ways—Petronel outgrowing all her childhood's promise and charm, Alison developing into a fascinating, vivid personality around whom the life and interest of the story centre from the first moment that she appears on the scene. The hero, Dr. Jim Cary, and his rather irritating fiancée, Lavinia Garland, are well drawn, consistent delineations.

The story opens with a pretty picture of Petronel as a child. Her mother, Lady Merrivale, has asked George Lumsden, artist, to come down and paint a portrait of Petronel. "'Come here and speak to the gentleman who is going to draw and paint you,' said her mother, persuasively. 'I don't want to be drawn and painted,' answered the little daughter, gravely. The artist who had come all the way from London stood still and smiled. The little brown shoes and stockings, the crumpled holland pinafore and big sun bonnet were just what he would have dressed her in himself; and the sweet flower face, with its sky-blue eyes, seemed to be hiding itself within the white calico petals from the too-inquisitive gaze of any outsiders. . . . The child stood in the sloping wood with her pinafore full of the bluebells she had gathered. Bright sunshine flecked the grass all round her, and the colour of the wild hyacinths lay like blue smoke all down the hillside. Up between the trees the sky showed deep and clear, and far away, in glimpses here and there, lay the blue sea. But Petronel's eyes were the purest bit of blue even in that landscape."

Lady Merrivale's views on country life amid the wilds of Devonshire differ considerably from those of the artist.

"'I am counting the days for the Easter recess to be over, so that we can go back to town. . . . Here we are wasting a whole splendid piece of April, and even a week in May, in this out-of-the-way place. . . . It's so tiresome of Dr. Cary not letting me take the children up to town with me.' 'London is not very nice for children,' said Lumsden. "I suppose not; I cannot see why. Our house overlooks the park.' 'Still, the park is not Devonshire.' 'No, thank goodness!' exclaimed Lady Merrivale, fervently." Flowers growing in uncongenial soil fade, or run to waste, and poor Petronel falls later, for the want of proper home training, from the fair promise of her childhood.

Dr. Cary and George Lumsden are old friends, and from Lady Merrivale he hears of Cary's engagement to Lavinia Garland. Lady Merrivale objects very much to the arrangement. "'He has behaved very like a goose in getting engaged to Lavinia Garland; but I knew how it would be when he became Dr. Garland's partner.' 'What is the girl like?' inquired George. 'Not much like a girl. Not that she is much more than thirty, but she has the primmest, most old-maidish ways I ever saw.' 'What made him become engaged to her? Is he in love with her?' 'Not he, and its all rubbish if he says he is. . . . Anyhow they drifted into an engagement, a folly which would never have happened if Jim had gone to sea, as all his fathers did before him.'"

Petronel suggests that George and she should pay a visit to Lavinia, "who always loves people, even people with big noses." George has rather a big nose, which Petronel asks him to cheer up under. "'Never mind,' she said, kindly; 'you couldn't help it. But I'm afraid you don't like it.' (George and she were great friends by this time.) 'I do like it,' she hastened to explain with crimson cheeks, but I'm very glad it isn't any bigger.'"

They reach the "dear gabled homestead, with a little wicket gate, and a huge garden sloping down to the meadow where the tiny river ran. . . . Beyond rose a hill from whence the country round Barnscombe could be seen, bounded by the blue Atlantic on the west." George's verdict of his friend's fiancée was brief but decisive. "She is pretty, womanly, and sweet, but she will never hold Jim."

When Jim had asked Lavinia to marry him (and many pages are devoted to explaining the causes that brought this about, when six words would have sufficed if they could have been said, viz., that "he was in love with her"), pity, not love, was the moving element that brought about the declaration. "He hoped also to find in her an ideal wife. . . . He actually imagined that men married girls because they possess certain attributes. . . . He did not know then that a man meets his ideal woman ready-made. He can hardly tell what her attributes are, and never thinks about what she might grow into, because he knows she is perfect as she is."

The engagement drifts on. Lavinia, who lived very much in awe of her mother, is distracted between an exaggerated sense of "duty" to this rather arbitrary old lady and devotion to her lover. He was growing weary of the constant reference to Mrs. Garland's wishes, placed first always in Lavinia's decisions. "Jim Cary was a very human man, and this constant introduction of his future mother-in-law's authority into all his suggestions he found decidedly irritating. He rather liked Mrs. Garland, in her proper place . . . but he did not like the way in which Lavinia placed his wishes second in her estimation, while her mother's whims were first."

Matters are in this strained condition when Mrs. Garland expresses her opinion on the subject of a man's susceptibility to improvement in the hands of a "capable" woman. "My dear, I never lost a day without trying to improve your poor father and he was faulty to the end. Men take a lot of improving, and in most cases it's trouble thrown away." This was in reply to one of Lavinia's remarks, "I find James a much more congenial companion than I used. . . . I cannot help feeling that he is improved" (and then follows one of those platitudes which make her speeches so irritating), "though of course it is presumptuous of me even to think that James could be improved."

Little wonder when Alison appeared on the scene with her bright freshness, her beauty and intelligence, her scorn for the petty standards which ruled her grandmother and Aunt Lavinia, little wonder, indeed, from that day when Jim Cary met her at the little wayside station on her arrival at Barnscombe till the close of the story, that she dominates the quiet circle in which she makes her home. A more charming, lovable character has never been conceived, yet on no two subjects were she and her relatives at one. They could not understand how she had time to think out, and speak with a sweet seriousness beyond her years, on the deep things of life, and the reader will notice that on all occasions it is Dr. Jim who listens to, and unconsciously inspires, many of the delightful thoughts to which Alison gives utterance. How her advent into "a corner of the West" affects the development and dénouement of Dr. Jim's love affair we must leave people to discover in the pages of this charming romance, which we cordially recommend to them for perusal.

* "A Corner of the West." By Henrietta Fowler. (Publishers: Hutchinson and Co., London. 6s.)

### Everybody's Opinion.

[Correspondence on all subjects is invited, but we cannot in any way be responsible for the opinions expressed by our correspondents. No communication can be entertained if the name and address of the correspondent is not given, as a guarantee of good faith but not necessarily for publication, or unless one side of the paper only is written on.]

### WORKHOUSE NURSES AND THE BATHING OF PATIENTS.

"Fuzzy-Wuzzy" writes: All honour and praise to the nurse who had the courage to refuse to bathe a male patient or to be present during the performance. I am sure all nurses will agree with me that it is not necessary for the nurse to be present. If she is within call should anything untoward happen it should be quite sufficient. Why should a nurse be asked to do such a thing? Because she is a nurse she is not lost to all sense of womanly feeling and delicacy. Nurses are often asked to do things contrary to all their womanly instincts, and sometimes, from a mistaken sense of a nurse's duty, they comply. Of course, in a case of real necessity or of life and death no true woman would hesitate while she asked herself whether her patient was male or female. If prompt measures had to be taken she would take them at once, and her patient would never forget or lose his respect for her. But the case under discussion does not come under that category. I sincerely wish that all nurses would combine and have the courage to refuse to do things against which all womanly instinct rebels. The feelings must get blunted in time, and all fine sense of delicacy lost unless a nurse makes a firm stand in the beginning and adheres to her resolution through thick and thin. I am not speaking as an outsider. I am a nurse of seven years' standing, six of which have been spent in hospital, and I must say none of our feelings in that hospital have been outraged. This, I think, is due in a great measure to our dear matron, who showed us where our duty began and ended. I, and I am sure every womanly nurse sympathises with, and has the sincerest admiration for, Nurse Norton. I heartily wish her success in soon getting a post more worthy of her, and I trust that her conduct will be an example to every nurse in doubt.

### DISTRICT NURSES.

Miss E. Hills, District Nurse, Borough Green, near Sevenoaks, writes: I have read with interest the different letters of private nurses, and, being intimate with several private nurses, I was told the other day that the opinions expressed in your columns have been a help to them in making things a little more agreeable in many ways. Before I came here I had furnished rooms found me. I get exactly the same salary here as I did then, but I cannot induce the committee to see the need of a hardworking nurse having a little home of her own instead of being in lodgings. A nurse has not the privilege of doing little things for herself in her spare time in lodgings, and my opinion is that no committee should bring a nurse into a place without first seeing their way clear to make provision for a comfortable home for her. I have been here just over a year, and I thought that if I proved to the ladies in my parish that their sick would benefit and appreciate my services they would provide me with one. A cottage might soon be made comfortable with little expense to nursing fund, which would gain in the end, because a nurse would stay longer if she could feel more settled. I shall be glad if you will kindly insert this in "Everybody's Opinion," in order that I may be guided by the comments it furnishes before deciding to resign. My salary is not sufficient to enable me to board and lodge myself, and I am quite alone in the world as regards relatives to help me. This is a very large district, and I get no time to do anything for myself as others may. I should be very sorry to leave, and believe if the committee had it put to them in the right light they would do something for me, especially as I have a little orphan sister who would live with me and help me.

### LARGE AND SMALL HOSPITALS.

"Thistle" writes: Your correspondent "Lily" makes some sweeping assertions regarding the training of nurses in large hospitals, and of course intimates the fact that she was trained in a small one, and therefore has a better knowledge of her duties as a nurse, and belongs to a "different class" from those trained in a large institution. Now this is not the case, as the training nurses receive nowadays and the examinations they have to pass prove, and must not be compared with the training "Lily" received perhaps twenty years ago. Medical men have their own reputation to uphold in respect to the proficiency certificates granted by them. She also makes a very serious charge against the character of the nurses employed in fever hospitals, stating (1) they care nothing for the comfort of their patients; (2) they do not tell the truth; (3) pay no attention to the orders of the doctor in regard to diet, &c., and charges them with cruelty to patients suffering from bedsores. This, I think, shows clearly that "Lily," if she is a nurse, is a very ignorant one to talk in such a manner, as it is one of the first duties taught in our Scotch hospitals that strict obedience to doctor's orders is the first requisite of an efficient nurse. I am engaged in one of the largest hospitals in Great Britain, at present treating over nine hundred patients, and having a staff of nurses numbering one hundred and fifty. With us bedsores are rare, as during our course of lectures one of the principal subjects taken up by the lecturer is "The Prevention of Bedsores" (not the treatment), and the precautions to be used learned there are, I can assure "Lily," faithfully carried out in the various wards. Of course, we occasionally get patients sent in to us from the surgical wards of some of the smaller hospitals suffering from bedsores, which are treated according to medical orders, combined with extreme care and kindness from the nurses on duty. As for the statement that nurses in fever hospitals don't speak the truth, I emphatically deny the accusation, and can only treat it with the contempt it deserves. But I would ask "Lily," before she again favours us with her opinions, to use a little discretion and gain a little more knowledge concerning fever nursing and nurses.

### COTTAGE HOSPITAL MANAGEMENT.

"An Hon. Member of Committee" writes: I was very much interested in reading the paragraph on "Cottage Hospital Management" in a recent issue of your paper, and only wish there was as considerate a committee of management for the Memorial Hospital in our little town. There is only a nurse-matron to manage (at the present time) three helpless cases and one convalescent, with cooking, housekeeping, laundry, and visitors, &c., besides the actual nursing and its numerous attendant duties. She has the help of a good general servant, and a laundress two days weekly. The hospital contains five beds and two cots in two large wards upstairs, and emergency ward and operation room (combined) downstairs, and being two storeys high it entails continuous running up and down. The other rooms upstairs are matron's and servant's bedrooms, bath-room, tank-room, and lavatory. Downstairs, operation-room, committee-room, kitchen, lavatory, and sitting-room, with laundry, mortuary, and out-houses, &c. The present matron is a native of the town, who applied for the appointment ten months ago, but who has now resigned her position, as her services are not appreciated, although she has worked hard and well, improved the place and the servant, and has by her economy and good management cleared the hospital of debt. She has nursed successfully twenty-six cases, with seven operations, single-handed, with no deaths, and I have known her to have five beds occupied for ten days at a stretch, and four beds occupied for twenty-one days at a time, but no matter how many or trying the cases she is never allowed trained or other assistance, except the cottage nurse for an odd night after an operation. The committee find fault, and say she is out too much, but considering she sometimes stays in for days together, and then only pays a flying visit to do errands in the town, we outsiders think it most inconsiderate and unjust, as they refuse her a probationer and yet object to the patients being left to the care of the servant, so what is the poor thing to do? For the past week she has had two accident cases with incessant diarrhœa, one, eight days downstairs, and the other seven days, for upstairs.

### For Reading to the Sick.

"THE Master is come, and calleth for thee."—*S. John* xi. 25.

"When thou passeth through the waters I will be with thee, and through the rivers, they shall not overflow thee."—*Isaiah* xliii. 2.

When some beloved voice that was to you
Both sound and sweetness, faileth suddenly,
And silence, against which you dare not cry,
Aches round you like a strong disease and new—
What hope! what help! what music will undo
That silence to your sense? Not friendship's sigh,
Not reason's subtle count . . . Nay, none of these!—
Speak Thou! availing Christ!—and fill this pause.

—*E. B. Browning.*

**Reading.**

The contemplation of the grievous sufferings, and the memory of the far greater pain and anguish borne by our dear Lord for us, and by many of His saints and servants for His sake, lead us to a right adjustment of our own sorrows, and often to a thankful realisation of their littleness. It is well for us to find the level, so to speak, of what we suffer; to remember how much more others have endured; to rest our souls upon the fact that the same Lord who has proved to be all-sufficient to the martyrs and saints of old can be, and is, the same now to us. Those who have gone before, where are they now? Do they watch us from above, as fellow warriors who have overcome in the same fight that we are now engaged in?

That wonderful "Cloud of Witnesses," which the writer of the Epistle to the Hebrews realised so vividly—how it has been added to and increased since he wrote his inspired words!

We have saints of our own, perhaps, who are gone out of the arena, crowned with laurel wreaths to await the coming of their Captain and our own!—mothers, fathers, sisters, brothers, friends, whose triumph over death we have seen with our own eyes—hear them speak to thee.

"Fear not," they say, those spirit warriors, "we have overcome; we sleep in Jesus: death was vanquished once and for all by Him; and now death serveth Christ, as messenger, to bring His own to be with Him in peace.

*E. A. D.*

We would see Jesus! for the shadows lengthen
Across this little landscape of our life—
We would see Jesus, our weak faith to strengthen
For the last weariness, the final strife!
We would see Jesus! for life's hand hath rested
With its dark touch upon both heart and brow,
And though our souls have many a billow breasted
Others are rising in the distance now.

We would see Jesus! other lights are paling,
Which for long years we have rejoiced to see;
The blessings of our pilgrimage are failing,
We would not mourn them, for we go to Thee.
We would see Jesus! yet the spirit lingers
Round the dear objects it has loved so long,
And earth from earth can scarce unclasp its fingers;
Our love to Thee makes not this love less strong.

We would see Jesus! Sense is all too blinding,
And Heaven appears too dim—too far away;
We would see Thee, Thyself our heart reminding
That Thou hast suffer'd—our great debt to pay.
We would see Jesus! This is all we're needing—
Strength, joy, and willingness come with the sight;
We would see Jesus—dying—risen—pleading!
Then Welcome Day, and farewell mortal night!

—*Anon.*

### Notes and Queries.

**The contents of the Editor's Letter-box have now reached such unwieldy proportions that it has become necessary to establish a hard and fast rule regarding Answers to Correspondents. In future, all questions requiring replies will continue to be answered in this column without any fee. If an answer is required by letter, a fee of half-a-crown must be enclosed with the note containing the enquiry. We are always pleased to help our numerous correspondents to the fullest extent, and we can trust them to sympathise in the overwhelming amount of writing which makes the new rules a necessity.**

**Every communication must be accompanied by the writer's name and address, otherwise it will receive no attention.**

*Training.*

(70) I am very anxious to be trained for a nurse. I have only one eye and wear glasses. The other eye is good, having almost double strength. I have good health, and I am very strong. Will any nurse or matron kindly tell me if I could get into an hospital or nursing home as a working probationer? If so, to what kind of hospital will be the best for applicant?—*Anxious One.*

Possibly you may be accepted as working probationer by one of the smaller workhouse infirmaries. The Northern Workhouse Nursing Association, Boston Arcade, Manchester, advertised lately in the "Mirror" for suitable probationers.

*Schools.*

(71) Will you kindly tell me if the Hospital for Chest Diseases, Brompton, London, also the Whiston Union Infirmary, Prescot, are proper training schools, and if the certificates given are recognised?—*Sister Clare.*

The certificates given at Brompton are for three years, and are recognised by the Local Government Board. You had better write about Whiston direct to the Local Government Board, Whitehall, S.W.

*Q.V.J.N.*

(72) Will you kindly tell me where I could get training as a Queen's nurse, and where I should apply? Also if I should require three years' training first?—*Anxious.*

You would require two years' hospital training first. The General Superintendent, Queen Victoria's Jubilee Nurses' Institute, Queen Katherine's Hospital, Regent's Park, N., will, however, be able to give you every information.

*Inebriates.*

(73) Mrs. L. would be greatly obliged if the editor of THE HOSPITAL would be so kind as to send her one or two addresses of homes for inebriates. She is most anxious to hear of a place of the sort which could be recommended in Scotland or the North of England. Mrs. L. has searched the advertising columns of THE HOSPITAL for months past, but could find no such advertisement, which seems strange. Surely there is need enough for them. She sends stamps for reply or particulars.

A fee of 2s. 6d. is charged for a private reply. There is a short list of such homes in "Burdett's Hospitals and Charities," but as the majority of homes are of a private character for one or two inmates, it is difficult to hear of them. An advertisement would probably bring a large number of replies.

*The Origin of Hospitals.*

(74) I take the liberty to ask you if you know of any small work on the origin of hospitals. 2. May I inquire if any of the London hospitals possess libraries for the use of the patients?—*T. H.*

There is no small book that we know of on the origin of hospitals, but you would probably find the information you want in a good encyclopædia. 2. Every hospital in London possesses a library for the patients' use.

*Training Home.*

(75) Would you kindly oblige me by forwarding one or two addresses of good training homes for nurses near town?—*F. H.*

A reply by post necessitates the query being accompanied by a fee of 2s. 6d. Most of the hospitals and larger workhouse infirmaries of the metropolis are training schools for nurses. For list see "The Nursing Profession: How and Where to Train," price 2s., from the Scientific Press.

*Linoleum.*

(76) Can you please give me the address where Staines' Linoleum may be procured? Is it possible to get linoleum cut to the exact size of a small room without joining? If not, which is the best way to have it laid so that dust may not accumulate underneath?—*Nurse H.*

Any of the large emporiums would supply the linoleum ready cut if the correct measurements are sent. The dust will not gather under it if the edges be exactly fitted and well tacked down.

*Pensions.*

(77) 1. Will you kindly give me some information about Princess Frederica's Homes? To whom could a lady apply for admission? 2. Could you kindly inform me of any hospitals or infirmaries which grant pensions to nurses? 3. Is any fully certificated nurse after 60 years of age entitled to the Junius S. Morgan Benevolent Fund?—*Nurse A.*

1. Write for particulars to the Secretary, the Princess Frederica's Homes for Gentlewomen, 6 and 7, Trinity Road, Tulse Hill, S.E. 2. The only charity for nurses is the Trained Nurses' Annuity Fund, 72, Cheapside, E.C. 3. No nurse is entitled to benefit by the Junius S. Morgan Fund. Each case is decided on its merits, preference being given to members of the Royal National Pension Fund.

*Address.*

(78) Will you kindly give the address of the office of the Central Bureau for Employment of Women, referred to in the article, "What Women Can Do," on page 353 of THE HOSPITAL of August 19th?—*Constant Reader.*

60, Chancery Lane

